# A spectral- topological network signature of drug-resistant epilepsy: a phase 1–2 study on resting-state EEG-based diagnostic biomarkers of drug resistance

**DOI:** 10.1016/j.nicl.2026.104016

**Published:** 2026-05-27

**Authors:** B.M. Sancetta, J. Lanzone, M.A.G. Matarrese, G. Lippa, M. Mesta, L. Ricci, M. Sferruzzi, S.P. Carbone, L. Veronese, G. Conti, M. Brunetti, F. Zappasodi, V. Di Lazzaro, M. Tombini, G. Assenza

**Affiliations:** a Research Unit of Neurology, Department of Medicine and Surgery, Università Campus Bio-Medico di Roma, Via Alvaro del Portillo 21, 00128 Rome, Italy; b Neurophysiology Service and Neurology Unit, IRCCS San Raffaele Scientific Institute, Via Olgettina, 60, 20132 Milan, Italy; c Research Unit of Intelligent Technologies for Health and Wellbeing, Department of Engineering, Università Campus Bio-Medico di Roma, Via Alvaro del Portillo 21, 00128 Rome, Italy; d Operative Research Unit of Neurology, Fondazione Policlinico Universitario Campus Bio-Medico, Via Alvaro del Portillo, 200, 00128 Roma, Italy; e Department of Neuroscience, Imaging and Clinical Sciences, Università degli Studi ‘G. d’Annunzio’ di Chieti-Pescara, Via dei Vestini, 66100 Chieti, Italy; f Institute for Advanced Biomedical Technologies, Università degli Studi ‘G. d’Annunzio’ di Chieti-Pescara, Via dei Vestini, 66100 Chieti, Italy; g Behavioral Imaging and Neural Dynamics Center, Università degli Studi ‘G. d’Annunzio’ di Chieti-Pescara, Via dei Vestini, 66100 Chieti, Italy

**Keywords:** Slow frequency activity, Functional EEG connectivity, Graph theory metrics, Drug-resistant epilepsy

## Abstract

**Objective:**

Drug-resistant epilepsy (DRE) is increasingly recognized as a disorder of large-scale brain networks. Here, we evaluated a candidate resting-state EEG-based biomarker for identifying DRE in a diagnostic context of use.

**Methods:**

We conducted a retrospective observational study (Phase 1–2 biomarker validation) on resting-state EEG recordings on healthy subjects (HS) and people with epilepsy (PwE). In PwE, EEGs were recorded after a second anti-seizure medication trial. The reference standard was longitudinal clinical outcome at 12 months (DRE vs. non-DRE). Index tests included the following quantitative EEG measures: spectral frequency-specific and aperiodic components, along with graph-theory metrics derived from the weighted phase-lag index. Multivariate logistic regression models assessed their discriminative value.

**Results:**

We enrolled 120 PwE (60 DRE) and 60 HS. DRE showed a distinct spectral profile, with increased δ (1–4 Hz) power, reduced α (8–12 Hz) power, and a steeper aperiodic slope compared with both HS and non-DRE. Network analysis revealed increased δ-band betweenness centrality and small-world index, alongside reduced global efficiency, indicating a shift toward a more regular and less integrative topology. These findings were independent of epilepsy etiology (p-values = 0.001–0.04). Adding EEG features significantly improved DRE classification compared with clinical variables alone (AUC: 0.83 ± 0.03 vs. 0.71 ± 0.02, p < 0.001).

**Conclusions:**

We revealed convergent spectral and network-level alterations that delineate an intrinsic network signature highly associated with DRE.

**Significance:**

Resting-state EEG metrics show promise as candidate diagnostic biomarkers for DRE, addressing an important unmet clinical need, though external validation is required.

## Highlights

• Drug-resistant epilepsy exhibits a cortical activity shift toward lower frequencies.
• Drug-resistant epilepsy is associated with a more regular brain network topology.
• Quantitative EEG metrics are strongly associated with drug-resistant condition.

## 1 Introduction

Epilepsy represents one of the most prevalent conditions of the central nervous system, defined by a persistent tendency to produce seizures (World Health Organization, 2019). In roughly one-third of people with epilepsy (PwE), seizure control remains insufficient despite optimized therapeutic approaches, resulting in a condition known as drug-resistant epilepsy (DRE) (Kwan et al., 2010). Drug-resistant epilepsy represents a major unmet clinical need, as current diagnostic pathways require prolonged therapeutic trials before confirmation, delaying effective interventions such as epilepsy surgery.

Over the past decades, research has advanced our understanding of epilepsy as a disorder of neural networks (Bartolomei et al., 2017). Even in the case of DRE, preclinical studies have supported the so-called neural network hypothesis of DRE (Fang et al., 2011), according to which seizure-related maladaptive remodelling of neural networks may contribute to anti-seizures medication (ASMs) failure. In this context, different quantitative frameworks on electroencephalography (EEG) have proven to be a valuable approach for both exploring the pathophysiological mechanisms underlying neural network alterations in epilepsy, such as hypersynchronization and imbalances in excitation and inhibition (Tóth et al., 2018). Moreover, preliminary quantitative EEG (qEEG)-based measures have shown promise as potential biomarkers (Califf, 2018) for predicting treatment response to both pharmacological and non-pharmacological therapies (Bodin et al., 2015, Lanzone et al., 2022, Lanzone et al., 2021, Pellegrino et al., 2018, Ricci et al., 2021, Routley et al., 2017).

Together, these foundational findings encouraged the present study, which aims to find a potential resting-state spectral–topological network signature characteristic of the DRE condition through qEEG analysis. In doing so, the study seeks to contribute to the discovery and preliminary validation of a potential EEG-based biomarker of DRE. The context of use of the present EEG biomarker in this study is primarily diagnostic, with the goal of identifying individuals at risk of developing drug resistance in the early treatment stages (that is, after the second ASM regimen). If our hypothesis were confirmed, this work could pave the way for validating new EEG-based biomarkers of DRE and for developing more personalized treatment approaches for PwE.

To reach this goal, we: (i) collected clinical data from a cohort of PwE who underwent clinical scalp EEG recordings (19 channels) following the titration completion of a second well-tolerated ASM; and (ii) evaluated qEEG metrics to characterize resting-state spectral and network changes linked to DRE. qEEG measures of PwE were also compared with those obtained from a group of age- and sex-matched healthy subjects (HS). Finally, we (iii) used cross-validated logistic regression models to compare the discriminative power of clinical and qEEG variables in differentiating our cohort according to the longitudinal clinical outcome at 12 months (reference standard).

## 2 Material and methods

This protocol received approval from the Ethics Committee (CET Lazio Area 2). All participants provided written informed consent before enrollment. Every procedure was conducted in accordance with the ethical principles outlined in the 1964 Declaration of Helsinki and its subsequent revisions.

### 2.1 Enrolment

**Fig. 1:**
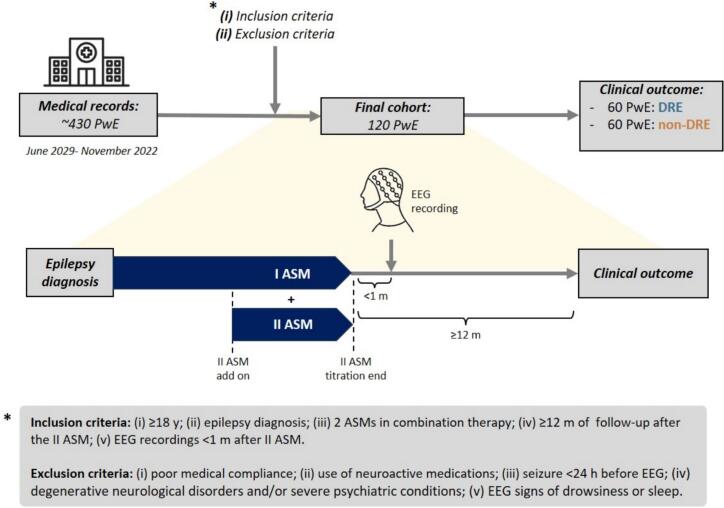
Flow diagram depicting the study design and cohort recruitment process.

We performed a retrospective, single-center observational study including cross-sectional EEG acquisition of the epilepsy outpatients service of Fondazione Policlinico Campus Bio-Medico (Rome). Recruitment lasted from January 2019 to November 2022 (see Fig. 1).

Inclusion criteria for selective PwE recruitment: (i) age ≥18 years; (ii) confirmed epilepsy diagnosis according to the current criteria of the International League Against Epilepsy (ILAE); (Fisher et al., 2005) (iii) exactly two ASMs trials administered in combination therapy at the time of EEG, both at the maximum effective and well-tolerated doses; (iv) at least 12 months of longitudinal clinical follow-up after the titration completion of the second ASM; (v) EEG recordings obtained within one month following the achievement of the maximal tolerated dose of the second ASM; (vi) a minimum of three minutes of resting-state EEG free of relevant artifacts; (vii) at least one brain magnetic resonance imaging (MRI) performed within the preceding five years.

Exclusion criteria for PwE: (i) poor medical compliance; (ii) concurrent use of neuroactive medications other than ASMs at the time of EEG; (iii) occurrence of seizure within 24 hours before EEG; (iv) history of progressive or degenerative neurological disorders and/or severe psychiatric conditions; (v) marked sleepiness before the EEG and/or EEG signs of drowsiness or sleep. The PwE cohort was dichotomized into DRE and non-DRE according to their clinical outcome 12 months after completion of the second ASM titration, based on the current ILAE definition of DRE and sustained seizure freedom, respectively (Kwan et al., 2010).

HS were included to contextualize disease-related alterations. Inclusion criteria of HS: (i) age between 18 and 65 years; (ii) no history of substance abuse, major medical illness, or psychiatric disorders; (iii) no current use of psychotropic or neuroactive agents; (iv) marked sleepiness before the EEG and/or EEG signs of drowsiness or sleep; (v) normal EEG activity as verified by experienced neurophysiologists.

### 2.2 Data collection

#### 2.2.1 Clinical data

We collected the following clinical variables known from the epilepsy literature to be associated with DRE (Hitiris et al., 2007):

(i) Demographic features/information related to epileptological history, that is female sex, current age, age at epilepsy onset, epilepsy duration, presence of interictal EEG abnormalities, both epileptiform (IEAs) and non-epileptiform (Hirsch et al., 2021), baseline seizure frequency (number of seizures occurring during the three months preceding the first ASM), focal onset epilepsy, and presence of multiple seizure types (Fisher et al., 2017);

(ii) Features related to epilepsy etiology, that is, the presence of structural etiology, neuroimaging abnormalities (both subcortical and/or cortical, even if deemed non-epileptogenic), extratemporal lobe epilepsy (TLE) or temporal lobe epilepsy (ETLE), inborn error of metabolism, and/or hippocampal sclerosis;

(iii) Features related to epilepsy treatment, that is, clinical response to the first ASM (≥50% reduction in seizure frequency following six months of well-tolerated and adequate dosage of the first ASM) (Kwan and Brodie, 2006).

Lastly, we documented detailed information regarding ASM therapy at the time of EEG acquisition, including the types and dosages administered.

#### 2.2.2 EEG acquisition

All EEGs were obtained with a 19-channel cap with a 32-channel Micromed system (SystemPlus software) based on the 10–20 electrode placement system (IFCN guidelines) (Nuwer et al., 1998) in the morning (same timeslot, 9.00–11.00 a.m.), during quiet rest with eyes closed in a sound-attenuated room with constant dimmed light, while seated in a reclined comfortable position. Impedance was <5 KΩ, reference on FPz, ground on FCz, sampling rate=256 Hz, A/D conversion=16 bits, and the pre-amplifier amplitude range=±3200 µV. Each EEG session lasted approximately 10–15 minutes.

### 2.3 EEG analysis

EEG preprocessing and analysis were performed blinded to clinical outcomes.

#### 2.3.1 EEG pre-processing

Offline EEG pre-processing was performed with EEGlab (Pernet et al., 2021), following the pipeline reported in the IPEG guidelines (Jobert et al., 2012): re-reference to a common average reference; 50 Hz notch filter and 0.5–70 Hz bandpass filter (Finite Impulse Response); visual inspection and manual rejection of huge artifacts and IEAs by experienced neurophysiologists, blind to clinical data and outcome; correction for pulse, eye-blink, and muscular artifacts using Independent Component Analysis supported by IClabel (Barbati et al., 2004) (each component labeled as an artifact by IClabel was visually checked through qualitative spectral and topography features before rejection) (Pion-Tonachini et al., 2019); artificial subspace reconstruction to remove high-amplitude artifacts (Plechawska-Wojcik et al., 2019). From the original raw signals, in accordance with current IFCN guidelines (Babiloni et al., 2020), we selected 180±25 seconds of continuous epochs, free from IEAs and relevant artifacts, for further analysis.

#### 2.3.2 Extraction of qEEG metrics

EEG analysis was carried out using the Brainstorm Toolbox implemented in MATLAB (Tadel et al., 2011). The index test consisted of the following qEEG measures: (i) power spectral density (PSD), (ii) aperiodic spectral components, (iii) functional connectivity and (iv) graph-theory parameters.

PSD (i) was estimated via a fast Fourier transform employing the Welch method (2-second windows with 50% overlapping windows) as a measure of cortical activity. The relative power (band power/total power) was computed for the following frequency ranges: δ (2–4 Hz), θ (5–7 Hz), α (8–12 Hz), and β (13–29 Hz), and it was preferred over absolute power as it provides greater stability for intra- and inter-subject comparisons. The γ band was excluded due to contamination by muscle-related artifacts in clinical EEG recordings, which compromise quantitative reliability. The spectral exponent and spectral offset were also derived to characterize the aperiodic component (ii) of the PSD (Donoghue et al., 2020).

For functional connectivity (iii), we employed the weighted Phase Lag Index (wPLI) to minimize the influence of volume conduction, a well-known confounding factor in EEG connectivity studies (Yoshinaga et al., 2020). Connectivity was assessed across the same frequency bands as used for PSD. Both PSD and wPLI values were computed for each EEG channel and then averaged to derive global measures of cortical activity and connectivity, respectively (Sancetta et al., 2022).

To explore network topology, wPLI matrices were binarized using a threshold corresponding to the 95th percentile of their cumulative probability distribution (Bullmore and Bassett, 2011). From these adjacency matrices, we extracted several graph-theory parameters (iv) consistent with previous literature: modularity, small-world index, betweenness centrality (BtwC), global efficiency (gE), clustering coefficient, and characteristic path length (λ) (Anastasiadou et al., 2019, Kaminski and Blinowska, 2018).

### 2.4 Statistical analysis

Statistical analysis was performed with Python 3.11. Potential missing data were handled using interpolation methods. The normality of data was checked via the Shapiro-Wilk test. Not-normally distributed data were reported by the median, range, and interquartile ranges (IQR). Normally distributed data were reported as mean value±sd. Categorical variables were compared via the χ^2^-test, while continuous variables were compared via a permutation test (10,000 iterations). Permutation results were expressed by reporting the most frequent p-values obtained after 100 permutations in order to provide results that are more stable and less affected by chance-related fluctuations (Cohen, 2014; Maris and Oostenveld, 2007; Nichols and Holmes, 2002); α level was set at 0.05.

To assess differences in spectral and connectivity measures between PwE and HS, we implemented mixed-model analyses of variance (ANOVA) with *Band* (four levels: δ, θ, α, β) as a within-subject factor and *Group* (two levels: HS vs. DRE or HS vs. non-DRE) as a between-subject factor. Comparisons between DRE and non-DRE groups were examined using a mixed-model ANOVA including *Group* (two levels: DRE, non-DRE) as the first between-subject factor and *structural brain abnormalities* (two levels: presence of structural brain abnormalities or pSBA and the absence of structural brain abnormalities or aSBA) or *focal epilepsy type* (two levels: TLE or ETLE) as second between-subject factors, applied separately for each frequency band.

The assumption of normality for ANOVA residuals was verified using Q–Q plots, and log transformations were applied when necessary. Mauchly’s test was used to examine sphericity, with Greenhouse–Geisser corrections applied as needed. Homogeneity of variances was assessed using Bartlett’s test. Post-hoc multiple comparison corrections were performed using the Benjamini–Hochberg false discovery rate method.

### 2.5 Logistic regression

A logistic regression analysis (maximum likelihood estimation approach) was conducted to evaluate and compare the ability of clinical variables and qEEG measures to classify PwE according to clinical outcome. The model included qEEG features that showed statistically significant differences between DRE and non-DRE. Logistic regression was executed twice: first using only clinical characteristics of DRE (REG_qEEG-_), and then using both clinical variables and qEEG metrics (REG_qEEG+_). Model performance was validated through a 5-fold cross-validation procedure, which was used to mitigate overfitting and estimate generalizability. Receiver operating characteristic (ROC) curves and the corresponding area under the curve (AUC) values were computed. For the REG_qEEG+_ model, performance was evaluated using AUC, sensitivity, specificity, positive predictive value (PPV), negative predictive value (NPV), accuracy (Acc), and F1-score. All metrics were reported as median ± standard deviation (sd). For each REG_qEEG+_ feature, the absolute values of the regression coefficients (β) and their respective p-values were provided to indicate each variable’s contribution to distinguishing DRE from non-DRE. Finally, permutation testing (10.000 iterations) was carried out for both REG_qEEG-_ and REG_qEEG+_ models to assess the significance of adding qEEG variables in differentiating the two groups.

## 3 Results

### 3.1 Clinical data

After revision of medical records, we enrolled 120 PwE and 60 HS (48.74±20.1 years old, 21 females). Among PwE, 60 PwE (namely, *DRE group*) were drug-resistant (46.7±22.4 years old, 36 females) while 60 PwE (namely, *non-DRE group*) achieved sustained seizure-freedom after follow-up (51.8±19.1 years old, 27 females). Detailed clinical details of our PwE cohort are displayed in Table 1.

**Table 1:** Clinical predictors of DRE and clinical data related to epilepsy treatment in aggregated form. *ASMs (anti-seizure medications); CBZ (carbamazepine); CNS (central nervous system); DRE (drug-resistant epilepsy); EEG (clinical standard electroencephalogram); ESL (eslicarbazepine acetate); ETLE (extratemporal lobe epilepsy); FA (focal onset aware seizures); FiA (focal onset with impairment of awareness seizures); FtB-TC (focal to bilateral tonic-clonic seizures); GM (motor generalized onset seizures); IQR (interquartile range); LCS (lacosamide); LEV (levetiracetam); LTG (lamotrigine); mm (months); mg (milligrams); MR (magnetic resonance); N (number); n.s. (non-significant); OXC (oxcarbazepine); PER (perampanel); PHB (phenobarbital); sd (standard deviation); TPM (topiramate); VPA (valproic acid); ZNS (zonisamide).*

**Clinical outcome**	**DRE**	**non-DRE**	
N, PwE	60	60	
**Clinical data**			**p-value**
**Demographic features**			
Sex, F/M ratio	36/24	27/33	n.s.
Age, years (mean ± sd)	46.7±22.4	50.6±18.9	n.s.
**Features related to medical history**			
Epilepsy onset, years (mean ± sd)	33.3 ± 24.2	38.8 ± 20.6	n.s.
Epilepsy duration, years (mean ± sd)	13.5 ± 12.7	12.5 ± 11.5	n.s.
Presence of EEG abnormalities, N (%)	60 (100%)	60 (100%)	-
Baseline seizure frequency, N/3 mos (median (range), IQR)	3 (1–65), IQR: 9.5	2 (1–42), IQR: 2.25	.005
Focal epilepsy, N (%)	54 (90%)	55 (91.7%)	n.s.
Types of seziures, N (median (range), IQR)	1 (1–3), IQR: 1	1 (1–2), IQR: 0	
- FA, N (%)	32 (53.3%)	29 (48.3%)	n.s.
- FiA, N (%)	29 (48.3%)	23 (38.3%)	n.s.
- FtB-TC, N (%)	17 (28.3%)	20 (33.3%)	n.s.
- GM, N (%)	3 (5%)	2 (3.3%)	n.s.
- GnM, N (%)	4 (6.7%)	3 (5%)	n.s.
**Features related to epilepsy etiology**			
Structural etiology, N (%)	26 (43.3%)	10 (16.7%)	.04
ETLE, N (%)	13 (21.7%)	16 (26.7%)	n.s.
Previous infection of CNS, N (%)	3 (5%)	0	n.s.
Autoimmune etiology, N (%)	0	2 (3.3%)	n.s.
Presence of neuroimaging abnormalities, N (%)	42 (70%)	34 (43.3%)	.05
Presence of inborn error of metabolism, N (%)	0	0	-
Presence of hippocampal sclerosis, N (%)	0	0	-
**Features related to epilepsy treatment**			
Response to first ASM, N (%)	11 (18.3%)	26 (43.3%)	.002
**Features related to epilepsy treatment**			
- LEV, N (%)	28 (46.7%)	30 (55.1%)	n.s.
Dosage, mg/die (median (range), IQR)	2500 (1250–3000), IQR: 1000	2500 (2000–3000), IQR: 1000	
- ESL, N (%)	22 (36.7%)	22 (37.9%)	n.s.
Dosage, mg/die (median (range), IQR)	1200 (800–1600), IQR: 300	800 (800–1200), IQR: 400	n.s.
- PER, N (%)	22 (36.7%)	16 (27.6%)	n.s.
Dosage, mg/die (median (range), IQR)	6 (4–10), IQR: 3.5	6 (2–10), IQR:1	n.s.
- LCS, N (%)	12 (20%)	10 (17.2%)	n.s.
Dosage, mg/die (median (range), IQR)	300 (250–400), IQR: 150	300 (200–400), IQR: 87.5	n.s.
- VPA, N (%)	10 (16.7%)	12 (20.7%)	n.s.
Dosage, mg/die (median (range), IQR)	1200 (400–2500), IQR: 1275	1200 (900–1500); IQR: 500	n.s.
- CBZ, N (%)	10 (16.7%)	6 (10.4%)	n.s.
Dosage, mg/die (median (range), IQR)	800 (600–1800), IQR: 600	800 (400–800), IQR: 300	n.s.
- ZNS, N (%)	2 (3.3%)	6 (10.3%)	n.s.
Dosage, mg/die (median (range), IQR)	200	200 (200–300), IQR: 75	n.s.
- TPM, N(%)	2 (3.3%)	6 (10.3%)	n.s.
Dosage, mg/die (median (range), IQR)	200	150 (100–400), IQR: 225	n.s.
- LMG, N(%)	4 (6.7%)	2 (3.4%)	n.s.
Dosage, mg/die (median (range), IQR)	150 (100–400), IQR: 225	400	n.s.
- OXC, N(%)	4 (6.7%)	1 (1.6%)	n.s.
Dosage, mg/die (median (range), IQR)	1400 (900–1900), IQR: 1000	-	-
- PHB, N (%)	1 (1.7%)	1 (1.7%)	n.s.
Dosage, mg/die	100	150	n.s.

### 3.2 Comparison between PwE and HS

#### 3.2.1 Spectral metrics

**Fig. 2:**
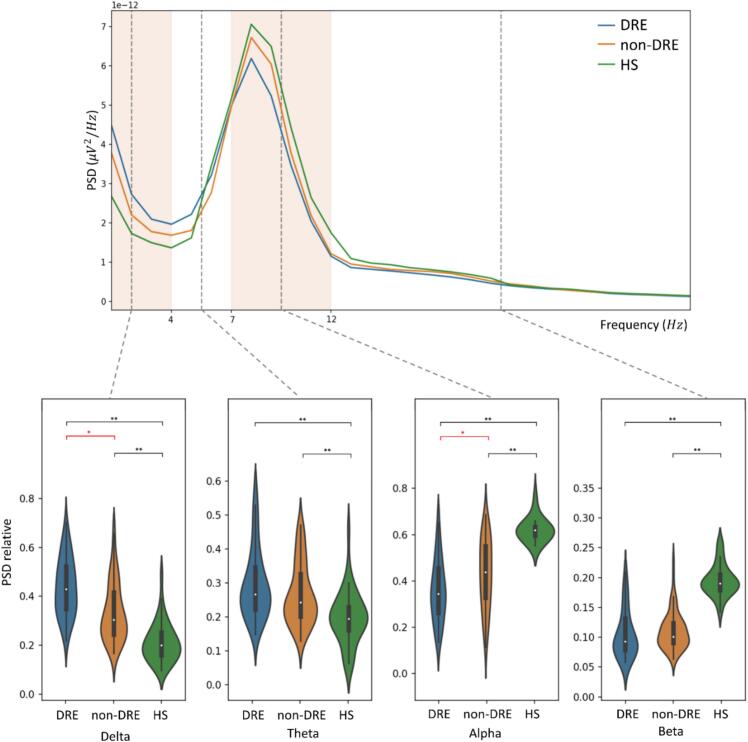
The superior panel depicts the mean PSDs (across-channels and across-subject average) of the three groups (DRE in *blue*, non-DRE in *orange* and HS in *green*). Red squares in transparency represent the frequency bands with a statistically significant difference between DRE and non-DRE. The inferior panel represents boxplots of relative PSD for each analyzed frequency band. As shown by PSD and boxplots, the DRE group exhibits higher absolute and relative delta power and lower absolute and relative alpha power than the non-DRE group. PwE had higher low-frequency power (delta and theta) and lower high-frequency power (alpha and beta) than HS. **Legend:** DRE (drug-resistant epilepsy); HS (healthy subjects); PSD (power spectral density); * (p-value <.05); ** (p-value <.001).

**Fig. 3:**
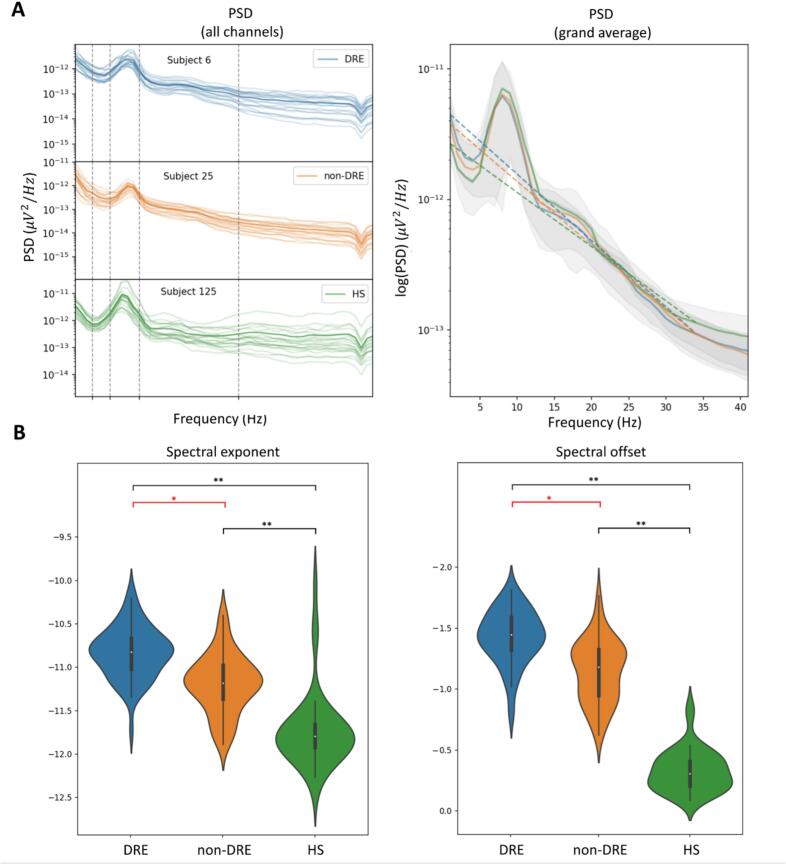
**Panel A**. On the left, overlapping PSD of all channels of three subjects from DRE (in *blue*), non-DRE group (in *orange*) and HS (in *green*). On the right, the grand-average of the mean PSD across the three groups in logarithmic scale; as shown in the picture, the DRE group has a steeper spectral decay (that is, a steeper slope of the PSD in double-logarithmic scale, here in dotted line) and a higher intercept compared to non-DRE and HS. As shown by the boxplots in **Panel B**, this evidence reflects on a more negative spectral exponent of DRE vs. non-DRE and DRE vs. HS (on the left) and a more negative spectral offset of DRE vs. non-DRE and DRE vs. HS (on the right). ***Legend:***DRE (drug-resistant epilepsy); HS (healthy subjects); PSD (power spectral density); * (p-value <.05); ** (p-value <.001).

ANOVA on PSD revealed a significant main *Group* effect (HS vs. DRE*:* F=170.04; HS vs. non-DRE: F=154.03, p-values <0.001) and a Group*Band interaction (HS vs. DRE*:* F=51.48; HS vs. non-DRE: F=38.72; p-values <0.001). Post-hoc analysis showed that PwE exhibited higher δ and θ-PSD and lower α and β-PSD than PwE (p-values <0.001, see Fig. 2). PwE also exhibited a more negative spectral exponent and spectral offset compared to HS (p-values <.001, see Fig. 3).

#### 3.2.2 Connectivity metrics

ANOVA revealed: (i) a significant main *Group* effect (HS vs. DRE: F=629.74, p-value <0.001; HS vs. non-DRE: F=533.26, p-value <0.001) and a significant Group*Band interaction (HS vs. DRE: F=67.99, HS vs. non-DRE: F=533.26, p-values <0.001) on wPLI; (ii) a significant main effect of *Group* both on gE (HS vs. DRE: F=78.40, p-value <0.001; HS vs. non-DRE: F=3.00, p-value=0.008) and BtwC (HS vs. DRE: F=84.32, HS vs. non-DRE: F=89.02, p-values <0.001); (iii) a significant Group*Band interaction on gE (HS vs. DRE: F=2.61, p-value=0.03; HS vs. non-DRE: F=3.26, p-value=0.04), BtwC (HS vs. DRE: F=3.51, p-value=0.02; HS vs. non-DRE: F=4.68, p-value=0.002), SWI (HS vs. DRE: F=3.62, p-value=0.01; HS vs. non-DRE: F=6.02, p-value=0.004) and λ (HS vs. DRE: F=2.66, p-value=0.03; HS vs. non-DRE: F=2.89, p-value=0.02).

**Fig. 4:**
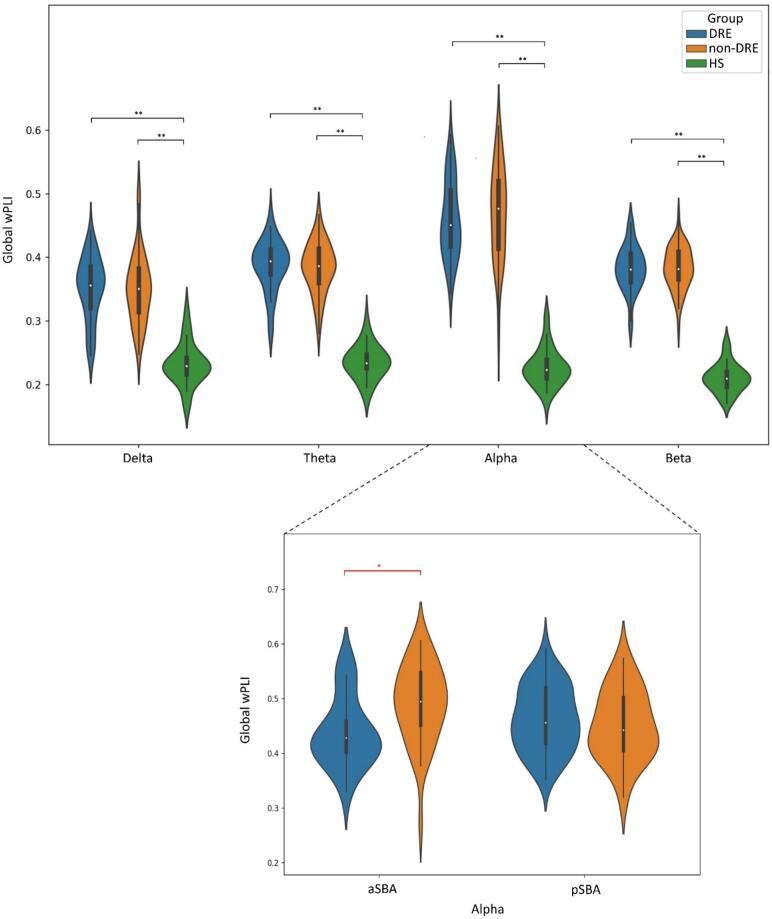
Results of post-hoc comparison on global wPLI. PwE (both DRE and non-DRE) had significantly higher global wPLI levels than HS for each analyzed frequency band. As shown in the inferior panel, ANOVA (between-subject factors: DRE vs. non-DRE, pSBA vs. aSBA) and post-hoc comparison revealed that DRE aSBA exhibited lower global alpha wPLI than non-DRE aSBA. ***Legend:***DRE (drug-resistant epilepsy); HS (healthy subjects); SBA (structural brain abnormalities); PwE (people with epilepsy); wPLI (weighted phase-lag index); * (p-value <.05); ** (p-value <.001).

**Fig. 5:**
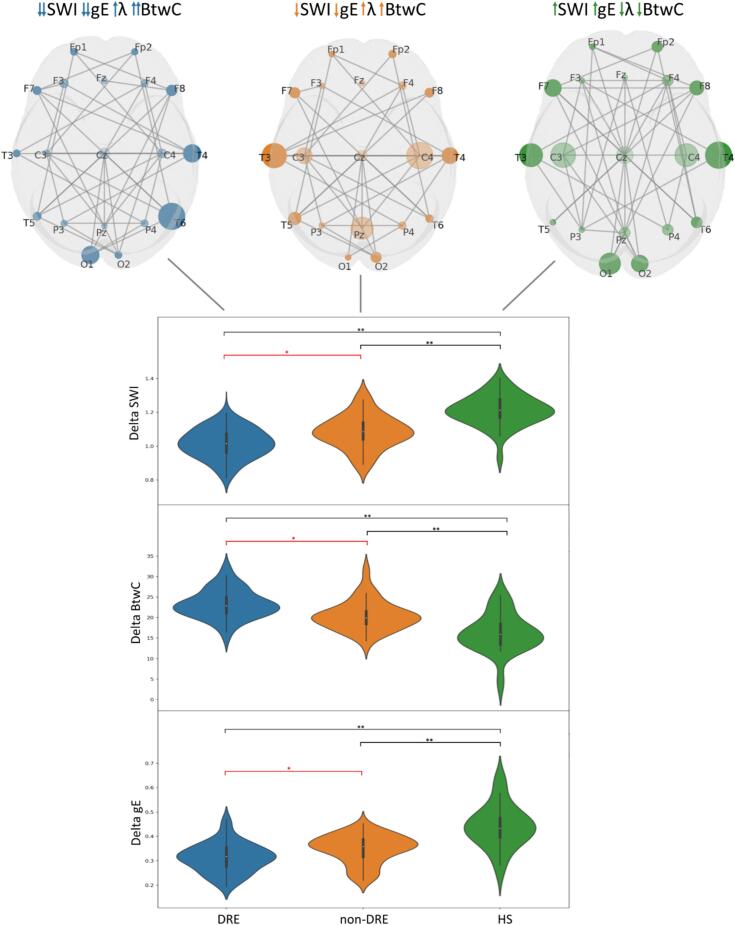
The superior panel displays the global networks (graph configuration after binarization of wPLI matrices) of one subject from the DRE group (*blue*), non-DRE group (*orange*) and HS group (*green*). The size of each electrode (node) is directly proportional to the BtwC level of each channel (relative magnitude compared to the maximum BtwC level of each graph configuration). Lines represent the edges connecting each node. Main results of ANOVA and post-hoc comparison on graph theory metrics are shown in the boxplots in the inferior panel. DRE exhibited lower SWI and gE and higher BtwC compared to non-DRE. All these findings, together with the highest λ of PwE vs. HS, suggest a network topology of DRE that deviates from a healthy small-world configuration (low SWI and high λ) with higher segmentation (high BtwC) and lower global integration (low gE). These characteristics are peculiar to regular networks. ***Legend:***BtwC (betweenness centrality); DRE (drug-resistant epilepsy); gE (global efficacy); HS (healthy subjects); SWI (small-word index); λ (characteristics path length); * (p-value <.05); ** (p-value <.001).

Post-hoc analysis showed that HS exhibited lower global wPLI than PwE (p-values <0.001, see Fig. 4), higher gE in δ, θ, and β frequency bands than PwE (p-value <0.001), lower δ-BtwC than DRE (p-value <0.001) and non-DRE (p-value=0.003), lower θ-BtwC than non-DRE (p-value=0.01), higher δ-SWI than DRE (p-value=0.009) and non-DRE (p-value=0.04), and longer δ-λ than DRE (p-value=0.02) and non-DRE (p-value=0.04). The main results of post-hoc analysis on graph theory metrics are reported in Fig. 5.

### 3.3 Comparison between DRE and non-DRE

#### 3.3.1 Spectral metrics

ANOVA revealed: (i) a significant main *Group* effect on δ-PSD (F=5.12, p-value=0.003), α-PSD (F=3.04, p-value=0.02); (ii) a significant Group*Structural brain abnormalities interaction on δ (F=2.05, p-value=0.03) and α-PSD (F=4.73, p-value=0.005); (*iii*) the absence of a significant main effect of factor *Structural brain abnormalities* on broadband spectral metrics.

**Fig. 6:**
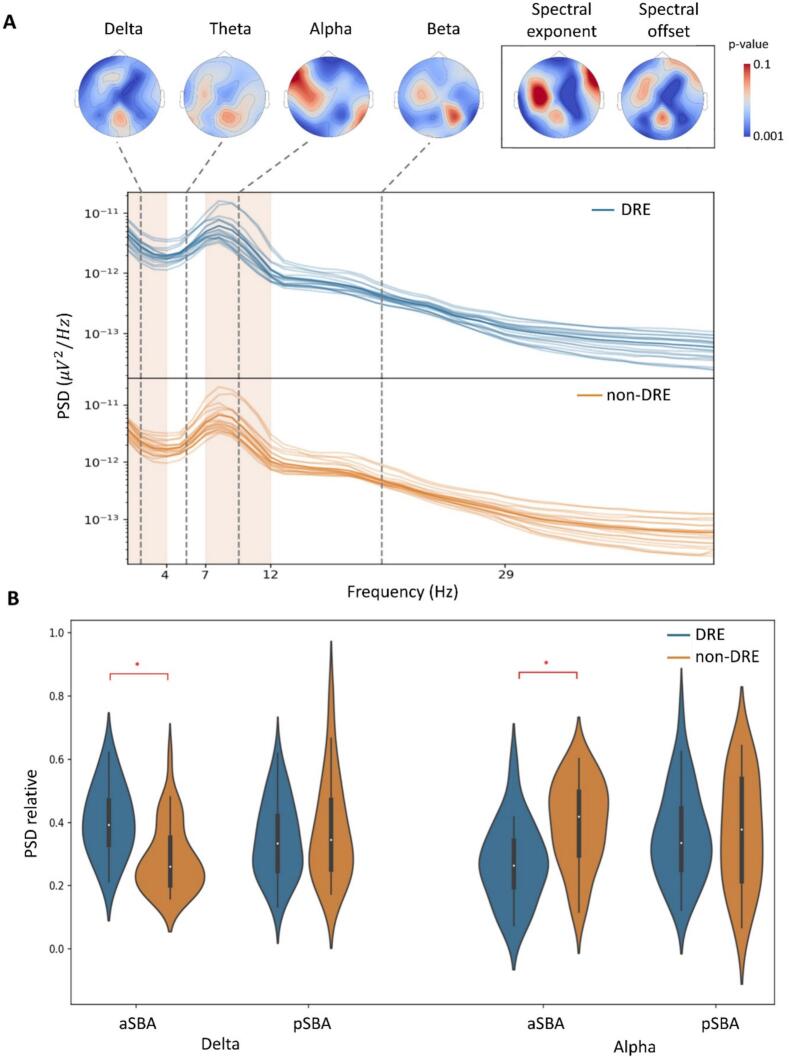
**Panel A.** Overlapping PSDs of each channel (across-subject mean) for the DRE group (in *blue*) and the non-DRE group (in *orange*). The scalp topography shows the significance map of the statistical difference between DRE and not-DRE for PSD, spectral offset and spectral exponent. Electrodes that show significant differences between DRE and non-DRE are dark blue. **Panel B.** The boxplots show the results of post-hoc analysis of ANOVA (between-subject factors: DRE vs. non-DRE, pSBA vs. aSBA) on delta and alpha PSD. ANOVA analysis confirmed the highest delta power and lower alpha power of DRE in ASBA. ***Legend:***ANOVA (analysis of variance); DRE (drug-resistant epilepsy); SBA (structural brain abnormalities); PSD (power spectral density); * (p-value <.05).

The overall DRE group showed higher δ-PSD (p-value=0.03) and lower α-PSD (p-value=0.04, see Fig. 2), as well as more negative exponent (p-value=0.02) and spectral offset (p-value=.002, see Fig. 3) than the overall non-DRE group. DRE aSBA exhibited higher δ (p-value=0.04) and lower α (p-value=0.05) relative power than non-DRE aSBA (see Fig. 6).

To further confirm these findings, we compared PSD, spectral exponents, and spectral offsets between DRE and non-DRE groups on scalp topography using a permutation test with multiple comparisons correction for the number of frequency bands and the number of electrodes. As shown in Fig. 6, the spectral differences in δ and α-PSD and spectral offset and exponent were significant in most of the channels with a widespread spatial distribution.

#### 3.3.2 Connectivity metrics

ANOVA analysis revealed: (i) a significant main *Group* effect (F=4.45, p-value=0.05) and a Group*Structural brain abnormalities interaction (F=7.72, p-value=0.006) on global α-wPLI; (ii) a significant main effect of *Group* on δ-gE (F=5.75, p-value=0.02), δ-BtwC (F=5.06, p-value=0.03), α-BtwC (F=3.58, p-value=0.04) and δ-SWI (F=4.04, p-value=0.03); (ii) a significant Group*Structural brain abnormalities interaction on α-BtwC (F=4.03, p-value=0.04); (*iii*) the absence of a significant main effect of factor *Structural brain abnormalities* on broadband spectral metrics.

Post-hoc analysis on global α-wPLI revealed that DRE aSBA exhibited lower global α-wPLI (p-value=0.008) than non-DRE aSBA (see Fig. 4)*.* As shown in Fig. 5*,* the overall DRE group showed lower δ-gE (p-value=0.04), higher δ-BtwC (p-value=0.007), and lower δ-SWI (p-value=0.02) than non-DRE. DRE aSBA exhibited higher α-BtwC (p-value=0.01) than non-DRE aSBA*.*

### 3.4 Logistic regression

**Fig. 7:**
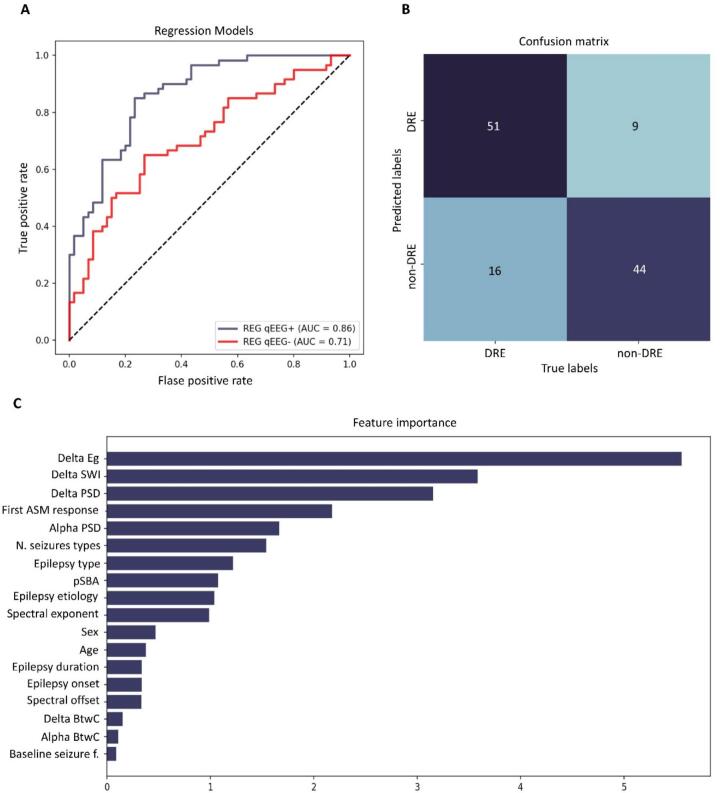
**Panel A.** ROC curves of logistic regression models (REG_qEEG+_ and REG_qEEG+_). For simplicity, we only displayed the mean ROC obtained after the cross-validation procedure. The addition of qEEG metrics to logistic regression bore significant added value to identifying people with DRE (p-value <.001). **Panel B.** Confusion matrix of the REG_qEEG+_ model (without cross-validation). **Panel C.** Linear coefficient (feature salience) of each feature imputed in the REG_qEEG+_ model; qEEG metrics contributed more to the model accuracy than clinical features. **Legend:***ASM (anti-seizure medications); AUC (Area Under the Curve); BtwC (betweenness centrality); DRE (drug-resistant epilepsy); f. (frequency); gE (global efficacy); pSBA (presence of structural brain abnormalities); N. (number); PSD (power spectral density); REGqEEG- (logistic regression without qEEG metrics); REGqEEG+ (logistic regression with qEEG metrics); ROC (Receiver Operating Characteristics).*

The main findings of logistic regression analysis are displayed in Fig. 7. REG_qEEG+_ was tuned with δ and α-PSD, spectral exponent and offset, α and δ-BtwC, δ-SWI, and δ-Eg, together with clinical features of DRE. ROC’s AUC of REG_qEEG-_ was 0.71±0.02 (p-value <0.001). The addition of qEEG features significantly improved the accuracy of the model (ROC’s AUC=0.86 ±0.03, p-value between RES_qEEG_- vs. RES_qEEG+_ <0.001). Performances of RES_qEEG+_ were the following: Sens=0.79±0.07, Spec=0.83±0.06, PPV=0.85±0.06, PPV=0.73±0.04, Acc=0.79±0.04 and F1-score=0.80±0.05. The five features of RES_qEEG+_ with the strongest influence on decision scores were, in descending order, δ-gE (β=5.56, p-value <0.001), δ-SWI (β=3.58, p-value <0.001), δ-PSD (β=3.16, p-value <0.001), response to first ASM (β=2.18, p-value <0.001), and α-PSD (β=1.67, p-value <0.001). Thus, spectral and graph theory metrics gave the most relevant contribution to RES_qEEG+_.

## 4 Discussions

### 4.1 Spectral slowing as a marker of epileptic activity in DRE

DRE showed distinct spectral characteristics compared to non-DRE, including elevated δ-PSD and reduced α-PSD (especially in aSBA subsample), together with a more negative spectral exponent and offset. In line with the network hypothesis of DRE pathophysiology, recurrent seizures are thought to trigger maladaptive neuroplastic processes and reorganization within brain networks (Boulenouar Mesraoua et al., 2023). Indeed, dynamic synaptic modifications, such as axonal sprouting and neuronal fiber loss, are well-established hallmarks of epileptogenesis (Chang and Lowenstein, 2003). These long-term potentiation-like mechanisms, coupled with alterations in the excitation–inhibition balance during ictal and interictal phases, may promote excessive synchronization at lower frequencies, resulting in enhanced δ (slow-wave) activity and a steeper (more negative) PSD slope.

Slow EEG oscillations are often regarded as an “epiphenomenon” reflecting the ongoing epileptogenic activity within the neocortex (Gambardella et al., 1995). Consequently, the δ band has been proposed by several authors as a potential scalp EEG biomarker of the epileptic network (De Stefano et al., 2022). The spectral exponent is also closely associated with EEG slowing: as it becomes more negative, the relative amplitude of higher frequencies diminishes compared to lower ones, indicating that a slower EEG corresponds to a sharper PSD decline (Colombo et al., 2019). Beyond the general notion of “EEG slowing,” the PSD slope has been linked to the functional equilibrium between excitatory and inhibitory neuronal activity. From this standpoint, the elevated δ-PSD and the more negative broadband spectral exponent observed in PwE (particularly among people with DRE) may reflect aberrant plasticity and long-term potentiation processes driven by epileptic network activity.

Conversely, α oscillations represent the dominant physiological rhythm in awake individuals, and their attenuation is a common feature of numerous neurological conditions, including cognitive deficits (Babiloni et al., 2007, Klimesch, 1999). The specific spectral profile identified in this study (namely decreased α-PSD and wPLI alongside increased δ-PSD in DRE) further supports the notion of a predominance of pathological (epileptic) network activity over physiological cortical activity in DRE. Alternatively, α-band reductions may be indicative of cognitive deficits (more frequent in DRE) (Vingerhoets, 2006) or of the mere “ongoing” cortical activation induced by subtle epileptic activity. However, this particular aspect cannot be conclusively determined within the constraints of the present study design.

Diffuse background slowing in PwE has traditionally been interpreted as a nonspecific sign of encephalopathy, for instance, due to ASMs' effects (Duncan, 1987). Our findings are consistent with this interpretation, showing reduced α and β-PSD and increased δ- and θ-PSD in PwE relative to HS. Notably, we also identified alterations in network topology (manifested by higher global wPLI, δ-BtwC, δ-λ, δ-SWI, and δ-Eg) in PwE compared to HS. These observations likely reflect, once again, the predominance of epileptic network dysfunction over physiological networks in PwE.

### 4.2 Alterations of resting-state network topology in DRE

DRE demonstrated significant alterations in network topology, characterized by increased δ-BtwC and decreased δ-Eg and δ-SWI compared to both non-DRE and HS. Evidence from invasive electrophysiological recordings strongly supports the notion that epilepsy represents a disorder of brain networks (Englot et al., 2016), wherein the epileptogenic zone forms a pathological network with well-defined spatial and functional hierarchies (Bartolomei et al., 2017). This aberrant organization disrupts cortical connectivity and function, thereby facilitating seizure recurrence. Within the framework of graph theory, the small-world configuration is recognized as the most efficient architecture, providing an optimal compromise between local specialization (segregation) and global communication (integration) (Bullmore and Bassett, 2011), which typifies the healthy brain network (Bernhardt et al., 2011). Regular networks display high local segregation (long λ) and reduced global integration (high clustering coefficient), whereas random networks show the opposite pattern (Watts and Strogatz, 2011). BtwC quantifies the degree of local segregation within the network, while gE assesses the capacity for parallel information transfer (Bazhanova et al., 2021).

From this perspective, the reduced SWI and gE, coupled with the elevated BtwC observed in the δ band among PwE (particularly in those with DRE), may indicate a shift of the epileptic network topology away from the optimal small-world configuration toward a more clustered, less integrated organization. Such a configuration may be less capable of supporting efficient parallel information flow. The increased λ detected in PwE could further reflect a more regular and less random network structure during resting-state activity, characterized by strong local connectivity but diminished global integration. These findings are consistent with prior graph-theory investigations of epilepsy, which have shown that epileptic networks tend to reorganize interictal background connectivity toward a more ordered topology that may facilitate seizure initiation (Adebimpe et al., 2015, Lee et al., 2017).

Collectively, these distinctive network abnormalities in DRE may represent the ability of epileptic circuits to reshape background network architecture into a more regular and less integrated form, potentially predisposing individuals with DRE to recurrent seizures despite appropriate therapeutic interventions. Nevertheless, determining whether this peculiar network configuration constitutes a cause or a consequence of persistent seizures remains uncertain within the current study design, underscoring the need for future investigations into the biological mechanisms underlying these observations.

### 4.3 Toward the identification of EEG-based diagnostic biomarkers of DRE

Our study corresponds to a phase 1–2 biomarker investigation according to the GREENBEAN criteria (Ewen et al., 2025), as it identifies candidate EEG-based biomarkers and provides preliminary estimates of classification performance without independent external validation. This topic addresses an important unmet clinical need in current clinical epilepsy, since, currently, no validated biomarkers exist for DRE, and its diagnosis still relies on the failure of successive ASMs trials.

Within this work, we found that the observed alterations in both spectral and network features were uniquely associated with the DRE condition itself, since they were not significantly influenced by major confounding factors commonly affecting qEEG interpretation, including the focal epilepsy type (as confirmed by ANOVA), the pharmacological load (both groups were treated with comparable ASM regimens at the time of EEG acquisition), and the epilepsy duration. Moreover, the majority of the network metrics extracted (especially those with the strongest influence on the regression model, that is, δ-gE and δ-SWI) were not influenced by the presence of structural brain abnormalities. For δ-PSD, subgroup analysis confirmed a statistically significant difference between DRE and non-DRE only in the MRI− group. In contrast, the lack of a significant difference between pSBA DRE and non-DRE patients may be explained by the intrinsically elevated δ power typically observed in individuals with underlying structural lesions, as widely reported in the literature (Gloor et al., 1977).

Crucially, our preliminary validation demonstrated the strong potential of spectral and connectivity EEG metrics to identify individuals at risk of developing drug resistance during the early stages of treatment history. Crucially, qEEG parameters contributed more substantially to the overall classification performance than traditional clinical predictors, reinforcing their potential clinical relevance.

We acknowledge that, based on our study design, our results are best interpreted within a diagnostic context of use, as they identify and predict individuals at risk of developing drug resistance in a population already exposed to two treatment attempts, rather than serving as a pre-treatment or purely prognostic biomarker. Nonetheless, the beginning of the second ASM trial is a clinically pivotal time point for epilepsy clinical management. First of all, the confirmation of DRE following adequate follow-up from the second ASM marks the beginning of the advanced diagnostic and therapeutic pathway for drug resistance (Yoo and Panov, 2019). Secondly, the likelihood of achieving seizure freedom with subsequent drug regimens decreases markedly after the second one (Chen et al., 2018), although multiple ASM trials are commonly tried in routine clinical practice. This results in delayed recognition of pharmaco-resistance, postponing appropriate interventions such as epilepsy surgery, and exposing PwE to ongoing seizures, repeated hospitalizations, and significant psychosocial burden (Luoni et al., 2011, Yoo and Panov, 2019). Therefore, the identification of diagnostic biomarkers of DRE in this early and crucial stage of treatment history (that is, the second ASM trial) could have substantial clinical utility by helping in DRE early recognition, by accelerating decision-making, saving years of illness and useful drug regimens, and reducing the burden of disease, therefore improving patient management and PwE’s quality of life.

Taken together, our findings provide preliminary but compelling evidence that EEG-based metrics may serve as promising diagnostic biomarkers of DRE, requiring phase 3-4 investigation studies. These results lay the groundwork for future research aimed at external validation of our findings and at clinical translation, with the ultimate goal to develop more personalized and targeted therapeutic strategies in epilepsy management. Importantly, in the future, the application of these EEG-based metrics could be extended to the time of epilepsy diagnosis (thereby serving as prognostic biomarkers), even though further studies with ad-hoc study designs are required to confirm our speculations.

## 5 Strengths and limitations

To the best of our knowledge, this is the first study to investigate qEEG metrics’ potential in delineating a specific electrophysiological phenotype of DRE using scalp EEG recordings. The current research framework is not without methodological challenges, foremost among them the issue of unequal feature distributions. Differences in the distribution of characteristics between DRE and non-DRE participants are inherent to the clinical context, reflecting fundamental clinical and pathophysiological distinctions between these groups. Such imbalances, if unaccounted for, could bias qEEG interpretations and yield findings that do not accurately represent true neurophysiological disparities. Our dataset offered a distinct advantage in this regard, as it maintained comparable class proportions between DRE and non-DRE for most clinically relevant variables that could confound qEEG interpretation (including the type, dosage, and number of ASMs) while also ensuring adequate sample size and representativeness.

Several limitations should be acknowledged, including the retrospective design, single-center recruitment, lack of external validation, and heterogeneity in seizure focus localization, all of which may introduce variability and bias. Notably, this heterogeneity partly stems from deliberate clinical selection criteria intended to reduce variability related to ASM number and type. Methodological constraints also relate to the EEG setup. The use of a conventional montage without subtemporal electrodes may have reduced sensitivity to temporal lobe activity and contributed to the lack of detectable effects of focal epilepsy type. We also recognize that high-density and intracranial EEG would provide a more precise assessment of network alterations in DRE, but their clinical use is limited by technical complexity, cost, and invasiveness. In contrast, standard 19-channel scalp EEG is well tolerated and offers greater feasibility, reproducibility, scalability, and cost-effectiveness, essential characteristics for potential translation into routine clinical practice.

Finally, although longer EEG recordings could provide more comprehensive sampling of brain activity, our analysis focused on stable, trait-like features of brain dynamics rather than transient events. Therefore, 3-minute multiple artifact-free, non-consecutive epochs represent a pragmatic balance, enabling reliable and reproducible estimates of spectral power and functional connectivity.

## 6 Conclusions

The present findings innovatively demonstrate that qEEG metrics can effectively characterize a specific intrinsic spectral and network configuration associated with DRE, therefore identifying a spectral–topological network signature of DRE, even when obtained through low-density scalp EEG. DRE was found to display a spectral displacement toward lower frequency ranges than non-DRE, potentially reflecting a greater degree of network dysfunction driven by the epileptic network. Moreover, the network topology associated with DRE showed a marked departure from the optimal small-world configuration, instead exhibiting a more regular and less globally integrated network topology. Importantly, the inclusion of qEEG indices notably enhanced the precision of DRE classification after a second ASM treatment regimen, contributing more substantially to its identification than traditional clinical variables. Collectively, these results provide a compelling basis for future research aimed at validating qEEG metrics as potential diagnostic biomarkers for DRE, with the ultimate goal of improving early identification and individualized therapeutic strategies, though external validation is required.

**Data and code availability statements**

Data and code may be provided to interested researchers upon reasonable request to the corresponding authors, after clearance from the local Ethics Committee.

**Funding**

This research received external funding from “PRIN: PROGETTI DI RICERCA DI RILEVANTE INTERESSE NAZIONALE – Bando 2022 PNRR Prot. P20225HWLZ. Research project title: EEG connectivity as an innovative biomarker to improve QUAlity of LIfe and The burden of disease in people with drug resistant epilepsY (EQUALITY)”.

## CRediT authorship contribution statement

**B.M. Sancetta:** Writing – review & editing, Writing – original draft, Methodology, Investigation, Formal analysis, Data curation. **J. Lanzone:** Writing – review & editing, Supervision, Formal analysis, Conceptualization. **M.A.G. Matarrese:** Data curation, Visualization, Writing – review & editing. **G. Lippa:** Writing – review & editing. **M. Mesta:** Writing – review & editing. **L. Ricci:** Writing – review & editing, Supervision, Resources. **M. Sferruzzi:** Writing – review & editing. **S.P. Carbone:** Writing – review & editing. **L. Veronese:** Writing – review & editing. **G. Conti:** Writing – review & editing. **M. Brunetti:** Writing – review & editing, Supervision, Funding acquisition. **F. Zappasodi:** Writing – review & editing, Supervision, Funding acquisition. **V. Di Lazzaro:** Writing – review & editing, Supervision, Funding acquisition. **M. Tombini:** Writing – review & editing, Visualization, Validation, Supervision, Resources, Investigation. **G. Assenza:** Writing – review & editing, Visualization, Validation, Supervision, Resources, Investigation, Funding acquisition, Data curation, Conceptualization.

## Declaration of competing interest

The authors declare that they have no known competing financial interests or personal relationships that could have appeared to influence the work reported in this paper.

## Data Availability

Data will be made available on request.

## References

[b0005] Adebimpe A., Aarabi A., Bourel-Ponchel E., Mahmoudzadeh M., Wallois F. (2015). Functional brain dysfunction in patients with benign childhood epilepsy as revealed by graph theory. PLoS One.

[b0010] Anastasiadou M.N., Christodoulakis M., Papathanasiou E.S., Papacostas S.S., Hadjipapas A., Mitsis G.D. (2019). Graph theoretical characteristics of EEG-based functional brain networks in patients with epilepsy: the effect of reference choice and volume conduction. Front. Neurosci..

[b0015] Babiloni C., Barry R.J., Başar E., Blinowska K.J., Cichocki A., Drinkenburg W.H.I.M. (2020). International Federation of Clinical Neurophysiology (IFCN) – EEG research workgroup: Recommendations on frequency and topographic analysis of resting state EEG rhythms. Part 1: Applications in clinical research studies. Clin. Neurophysiol..

[b0020] Babiloni C., Cassetta E., Binetti G., Tombini M., Del Percio C., Ferreri F. (2007). Resting EEG sources correlate with attentional span in mild cognitive impairment and Alzheimer’s disease. Eur. J. Neurosci..

[b0025] Barbati G., Porcaro C., Zappasodi F., Rossini P.M., Tecchio F. (2004). Optimization of an independent component analysis approach for artifact identification and removal in magnetoencephalographic signals. Clin. Neurophysiol..

[b0030] Bartolomei F., Lagarde S., Wendling F., McGonigal A., Jirsa V., Guye M. (2017). Defining epileptogenic networks: contribution of SEEG and signal analysis. Epilepsia.

[b0035] Bazhanova E.D., Kozlov A.A., Litovchenko A.V. (2021). Mechanisms of drug resistance in the pathogenesis of epilepsy: role of neuroinflammation. a literature review. Brain Sci..

[b0040] Bernhardt B.C., Chen Z., He Y., Evans A.C., Bernasconi N. (2011). Graph-theoretical analysis reveals disrupted small-world organization of cortical thickness correlation networks in temporal lobe epilepsy. Cereb. Cortex.

[b0045] Bodin C., Aubert S., Daquin G., Carron R., Scavarda D., McGonigal A. (2015). Responders to vagus nerve stimulation (VNS) in refractory epilepsy have reduced interictal cortical synchronicity on scalp EEG. Epilepsy Res..

[b0050] Boulenouar Mesraoua, Francesco Brigo, Simona Lattanzi, Bassel Abou-Khalil, Hassan Al Hail, Ali A. Asadi-Pooya. Drug-resistant epilepsy: Definition, pathophysiology, and management. J Neurol Sci 2023;452. 10.1016/j.jns.2023.120766 37597343

[b0055] Bullmore E.T., Bassett D.S. (2011). Brain graphs: graphical models of the human brain connectome. Annu. Rev. Clin. Psychol..

[b0060] Califf R.M. (2018). Biomarker definitions and their applications. Exp. Biol. Med..

[b0065] Chang B.S., Lowenstein D.H. (2003). Mechanisms of disease epilepsy. N. Engl. J. Med..

[b0070] Colombo M.A., Napolitani M., Boly M., Gosseries O., Casarotto S., Rosanova M. (2019). The spectral exponent of the resting EEG indexes the presence of consciousness during unresponsiveness induced by propofol, xenon, and ketamine. Neuroimage.

[b0075] Donoghue T., Haller M., Peterson E.J., Varma P., Sebastian P., Gao R. (2020). Parameterizing neural power spectra into periodic and aperiodic components. Nat. Neurosci..

[b0080] Duncan J.S. (1987). Antiepileptic drugs and the electroencephalogram. Epilepsia.

[b0085] Englot D.J., Konrad P.E., Morgan V.L. (2016). Regional and global connectivity disturbances in focal epilepsy, related neurocognitive sequelae, and potential mechanistic underpinnings. Epilepsia.

[b0090] Ewen J.B., Babiloni C., Collins G.S., Ethridge L.E., Gotman J., Ikeda A. (2025). The GREENBEAN checklist for reporting studies evaluating the effectiveness of EEG-based biomarkers. Clin. Neurophysiol..

[b0095] Fang M., Xi Z.Q., Wu Y., Wang X.F. (2011). A new hypothesis of drug refractory epilepsy: neural network hypothesis. Med. Hypotheses.

[b0100] Fisher R.S., Cross J.H., D’Souza C., French J.A., Haut S.R., Higurashi N. (2017). Instruction manual for the ILAE 2017 operational classification of seizure types. Epilepsia.

[b0105] Fisher R.S., Van Emde B.W., Blume W., Elger C., Genton P., Lee P. (2005). Epileptic seizures and epilepsy: Definitions proposed by the International League Against Epilepsy (ILAE) and the International Bureau for Epilepsy (IBE). Epilepsia.

[b0110] Gambardella A., Gotman J., Cendes F., Andermann F. (1995). Focal intermittent delta activity in patients with mesiotemporal atrophy: a reliable marker of the epileptogenic focus. Epilepsia.

[b0115] Hirsch L.J., Fong M.W.K., Leitinger M., Laroche S.M., Beniczky S., Abend N.S. (2021). American clinical neurophysiology society’s standardized critical care EEG terminology: 2021 version. J. Clin. Neurophysiol..

[b0120] Hitiris N., Mohanraj R., Norrie J., Sills G.J., Brodie M.J. (2007). Predictors of pharmacoresistant epilepsy. Epilepsy Res..

[b0125] Jobert M., Wilson F.J., Ruigt; G.S.F., Brunovsky M., Prichep L.S., Drinkenburg W.H.I.M. (2012). Guidelines for the recording and evaluation of pharmaco-eeg data in man: the international pharmaco-EEG society (IPEG): the IPEG pharmaco-EEG guideline committee. Neuropsychobiology.

[b0130] Kaminski M., Blinowska K.J. (2018). Is graph theoretical analysis a useful tool for quantification of connectivity obtained by means of EEG/MEG techniques?. Front. Neural Circuits.

[b0135] Klimesch W. (1999). EEG alpha and theta oscillations reflect cognitive and memory performance: a review and analysis. Brain Res. Rev..

[b0140] Kwan P., Arzimanoglou A., Berg A.T., Brodie M.J., Hauser W.A., Mathern G. (2010). Definition of drug resistant epilepsy: consensus proposal by the ad hoc task force of the ILAE commission on therapeutic strategies. Epilepsia.

[b0145] Kwan P., Brodie M.J. (2006). Refractory epilepsy: mechanisms and solutions. Expert Rev. Neurother..

[b0150] Lanzone J., Boscarino M., Tufo T., Di Lorenzo G., Ricci L., Colicchio G. (2022). Vagal nerve stimulation cycles alter EEG connectivity in drug-resistant epileptic patients: a study with graph theory metrics. Clin. Neurophysiol..

[b0155] Lanzone J., Ricci L., Tombini M., Boscarino M., Mecarelli O., Pulitano P. (2021). The effect of Perampanel on EEG spectral power and connectivity in patients with focal epilepsy. Clin. Neurophysiol..

[b0160] Lee C., Im C.H., Koo Y.S., Lim J.A., Kim T.J., Byun J.I. (2017). Altered network characteristics of spike-wave discharges in juvenile myoclonic epilepsy. Clin. EEG Neurosci..

[b0165] Luoni C., Bisulli F., Canevini M.P., De Sarro G., Fattore C., Galimberti C.A. (2011). Determinants of health-related quality of life in pharmacoresistant epilepsy: results from a large multicenter study of consecutively enrolled patients using validated quantitative assessments. Epilepsia.

[b0170] Maris E., Oostenveld R. (2007). Nonparametric statistical testing of EEG- and MEG-data. J. Neurosci. Methods.

[b0175] Nuwer M.R., Comi G., Emerson R., Fuglsang-Frederiksen A., Guerit M., Hinrichs H. (1998). IFCN standards for digital recording of clinical EEG. Electroencephalogr. Clin. Neurophysiol..

[b0180] Pellegrino G., Mecarelli O., Pulitano P., Tombini M., Ricci L., Lanzone J. (2018). Eslicarbazepine acetate modulates EEG activity and connectivity in focal epilepsy. Front. Neurol..

[b0185] Pernet C.R., Martinez-Cancino R., Truong D., Makeig S., Delorme A. (2021). From BIDS-formatted EEG data to sensor-space group results: a fully reproducible workflow with EEGLAB and LIMO EEG. Front. Neurosci..

[b0190] Pion-Tonachini L., Kreutz-Delgado K., Makeig S. (2019). The ICLabel dataset of electroencephalographic (EEG) independent component (IC) features. Data Brief.

[b0195] Plechawska-Wojcik, M., Kaczorowska, M., Zapala, D., 2019. The artifact subspace reconstruction (ASR) for EEG signal correction. A comparative study. Adv. Intelligent Syst. Comput., 853, https://doi.org/10.1007/978-3-319-99996-8_12.

[b0200] Ricci L., Assenza G., Pulitano P., Simonelli V., Vollero L., Lanzone J. (2021). Measuring the effects of first antiepileptic medication in temporal lobe epilepsy: predictive value of quantitative-EEG analysis. Clin. Neurophysiol..

[b0205] Routley B.C., Singh K.D., Hamandi K., Muthukumaraswamy S.D. (2017). The effects of AMPA receptor blockade on resting magnetoencephalography recordings. J. Psychopharmacol..

[b0210] Sancetta B.M., Ricci L., Assenza G., Boscarino M., Narducci F., Vico C. (2022). EPIAMNE: a new scoring system for differentiating transient EPIleptic AMNEsia from transient global amnesia. Brain Sci..

[b0215] De Stefano P., Carboni M., Marquis R., Spinelli L., Seeck M., Vulliemoz S. (2022). Increased delta power as a scalp marker of epileptic activity: a simultaneous scalp and intracranial electroencephalography study. Eur. J. Neurol..

[b0220] Tadel F., Baillet S., Mosher J.C., Pantazis D., Brainstorm L.RM. (2011). A user-friendly application for MEG/EEG analysis. Comput. Intell. Neurosci..

[b0225] Tóth K., Hofer K.T., Kandrács Á., Entz L., Bagó A., Erőss L. (2018). Hyperexcitability of the network contributes to synchronization processes in the human epileptic neocortex. J. Physiol..

[b0230] Vingerhoets G. (2006). Cognitive effects of seizures. Seizure.

[b0235] Watts, D.J., Strogatz, S.H., 2011. Collective dynamics of “small-world” networks. The Structure and Dynamics of Networks, vol. 9781400841356, https://doi.org/10.1515/9781400841356.301.

[b0240] World Health Organization. WHO | Epilepsy: a public health imperative. 2019.

[b0245] Yoo J.Y., Panov F. (2019). Identification and treatment of drug-resistant epilepsy. CONTINUUM Lifelong Learning in Neurology.

[b0250] Yoshinaga K., Matsuhashi M., Mima T., Fukuyama H., Takahashi R., Hanakawa T. (2020). Comparison of phase synchronization measures for identifying stimulus-induced functional connectivity in human magnetoencephalographic and simulated data. Front. Neurosci..

